# Preliminary Results Indicate That Inactivated Vaccine against Paratuberculosis Could Modify the Course of Experimental *Mycobacterium bovis* Infection in Calves

**DOI:** 10.3389/fvets.2017.00175

**Published:** 2017-10-18

**Authors:** Miriam Serrano, Natalia Elguezabal, Iker A. Sevilla, María V. Geijo, Elena Molina, Ramón A. Juste, Joseba M. Garrido

**Affiliations:** ^1^Animal Health Department, NEIKER-Instituto Vasco de Investigación y Desarrollo Agrario, Bizkaia Science and Technology Park, Derio, Spain

**Keywords:** paratuberculosis, bovine tuberculosis, vaccine, interference, crossed-protection

## Abstract

Although paratuberculosis (PTB) vaccination has been recognized as an effective tool to control the disease, its use has been limited in countries undergoing bovine tuberculosis (bTB) eradication programs because of its interference with the diagnostic techniques. Due to this restraint, little is known about the effect of vaccinating against PTB on the progression of bTB infection. To assess this topic, an experimental infection was carried out including the following three groups of five calves each: non-vaccinated infected with *Mycobacterium bovis* (NVI), vaccinated against PTB infected with *M. bovis* (VI), and vaccinated against PTB non-infected (VNI). The level of infection attending to pathological and bacteriological parameters was evaluated at necropsy in collected tissue samples. Infection was confirmed in all challenged animals being the lung and thoracic regions most affected for all studied parameters. The VI group presented 15.62% less gross lesions in the thoracic region than the NVI, although no significant differences were found. Only one vaccinated animal presented gross lesions in the lung, compared to three non-vaccinated calves. NVI animals showed an average of 1.8 lung lobes with gross lesions whereas in the vaccinated group the average number of affected lobes was 0.2, representing an 89% reduction. Significant differences were not found, although a tendency was observed (*p* = 0.126). Pathological and culture scores showed the same tendency. Vaccination induced a 71.42 and 60% reduction in lesion and culture scores in the lung as well as a 23.75 and 26.66% decline, respectively, in the thoracic region. The VI group showed lower positivity in the rest of the areas for all measured criteria except for the head. In order to reinforce our results, further research on a larger sample size is needed, but the results from this study suggest that PTB vaccination could confer certain degree of protection against bTB infection, supporting the view that PTB vaccination could increase resistance to the main mycobacterioses that affect animals.

## Introduction

Bovine tuberculosis (bTB) and paratuberculosis (PTB) are mycobacterial diseases that have a huge economic impact on cattle, especially on dairy herds (1, 2). Both present a widespread distribution, affecting domestic hosts (3, 4) and wildlife species (5, 6), promoting the successful dissemination of their etiological agents.

Paratuberculosis or Johne’s disease is caused by *Mycobacterium avium* subsp. *paratuberculosis* (MAP). Progression of the disease causes a chronic gastrointestinal granulomatous inflammation. Different factors, such as the long incubation period of the disease, fecal-oral route transmission, intermittent excretion periods added to high resistance of mycobacteria in the environment, and limited performance of diagnostic methods, make control of the infection difficult to achieve. PTB vaccination has been proven to restrain the disease in cattle (7), sheep (8), and goats (9). Excretion of bacterial burden is considerably reduced, containing the spread of the infection and therefore diminishing the number of clinical cases (10, 11). Nevertheless, the interference induced by PTB vaccination with the current interpretation criteria (12) of the bTB official diagnostic tests (13) results in the restricted use of PTB vaccination. MAP-based vaccination in cattle is not allowed by the Animal Health Authorities of most countries. Despite the diagnostic interference, a certain degree of containment of the lesion dissemination from the target organs after a bTB infection has been previously reported in PTB-vaccinated goats (14). This suggests that if interference issues are avoided, PTB vaccination can be used for PTB control and might also be beneficial against bTB conferring some degree of protection.

The degree of interference of PTB vaccination with official bTB diagnostic tests has been evaluated previously in cattle (13, 15) and goats (16, 17). Cross-reactivity in the skin test has proven to be limited if the comparative intradermal test is used in bTB-free bovine herds (13). These findings have been based on an exhaustive analysis of results from a vaccine clinical trial under field conditions. To evaluate the effect of PTB vaccination on bTB infection and the interference due with bTB diagnosis, an experimental infection with *Mycobacterium bovis* in bovines previously vaccinated against PTB was performed. Results on interference using alternative diagnostic criteria and specific antigens have been reported in a separate paper (12) while pathological and bacteriological changes associated with vaccination in a bTB experimental infection are the goal of this report.

## Materials and Methods

### Ethics Statement

Animals used in this study had their origin in commercial farms. With the purpose of obtaining data for this trial, calves were submitted to clinical practices standardized and regulated by the European, Spanish and Regional Law and Ethics Committee. The experimental design underwent ethical review and approval by NEIKER’s Animal Care and Use Committee and by the Agriculture Department (PARAPATO-1264-BFA).

### Animal Selection

Thirty Friesian calves from 13 different farms in northern Spain were selected in a feedlot. For final selection of animals, absence of previous contact with mycobacteria was considered. IFN-γ release assay (IGRA) with standard tuberculins (A-PPD, B-PPD) as well as with more specific antigens (ESAT-6/CFP10 and Rv3615c) (18) already tested for the diagnosis of bTB was the assay used for this purpose. The first blood sampling was carried out at week 0, at the age of 2 months. After the blood samples were collected, 20 randomly selected animals were vaccinated subcutaneously in the dewlap with 1 ml of a heat-inactivated vaccine (Silirum^®^ CZV, Pontevedra, Spain) and 10 remained unvaccinated. In order to reassure the absence of previous contact with *M. bovis* or other mycobacteria, blood samplings at the feedlot were repeated twice, at weeks 4 and 12. Finally 10 vaccinated and five non-vaccinated animals with negative results for IGRA and without evident pathologies were selected and transported to the biosafety level 3 (BSL-3) facilities in NEIKER where three groups of five animals each were formed.

### *M. bovis* Challenge

A 2-week adaptation period was established for the calves after their arrival at the BSL-3 facilities. At week 18 post-vaccination, five vaccinated and five non-vaccinated animals were challenged by the endotracheal route with 10^6^ colony-forming units (CFUs) of a *M*. *bovis* field isolate suspended in 2 ml of phosphate-buffered saline after intramuscular sedation with Xylazine (10 mg/50 kg). The *M. bovis* challenging isolate was spoligotype SB0339 according to the *M. bovis* Spoligotype Database website (http://www.mbovis.org). The final experimental groups were as follows: PTB vaccinated and *M. bovis* infected (VI), PTB vaccinated and *M. bovis* non-infected (VNI), and PTB non-vaccinated and *M. bovis* infected (NVI).

### Postmortem Studies

The animals were slaughtered at week 14 post-infection in three consecutive days, five calves per day. Upon sedation with XILAGESIC^®^ 2% (10 mg/50 kg lw) (Laboratorios Calier, S.A., Barcelona, Spain), animals were euthanized by an intravenous injection of T61 (4–6 ml/50 kg) (Intervet International GMBH, Unterschleissheim, Germany). Complete necropsy was performed although special focus was set on the respiratory system. All tissue specimens were individually collected and processed for pathological and microbiological analysis. Collected tissues per region included were: head [tonsils, nasal turbinate, and parotid and retropharyngeal and mandibular lymph nodes (LNs)], thorax (tracheal, tracheobronchial, mediastinal, pulmonary, and prescapular LNs), lung (right and left cranial and caudal lobes and medium and accessory lobes), abdomen (liver, spleen, and hepatic LNs), and others (prefemoral LNs).

### Gross Pathology

All tissues were visually inspected for lesions compatible with TB infection. Scoring of lesions according to Palmer et al. (19) was performed independently by two researchers, and the mean value of both scores was used. Briefly, the scoring system for lung was as follows: 0: no visible gross lesion, 1: no visible external gross lesion but internal detected after splicing, 2: less than five lesions smaller than 10 mm, 3: over five lesions smaller than 10 mm, 4: more than one lesion bigger than 10 mm, and 5: gross confluent lesions. In the case of LNs, scoring was as follows: 0: no visible gross lesions, 1: focal lesions of 1–2 mm, 2: a lot of small foci, and 3: extended lesions. Once scores were assigned to each lesion, total and regional scores per animal were calculated by adding them.

### Bacterial Culture

Bacterial tissue culture was performed as described previously (20). Briefly, 2 g of tissue samples were homogenized in 10 ml of sterile distilled water. Five milliliters of the suspension was decontaminated and processed for liquid culture in BBL MGIT tubes supplemented with BBL MGIT PANTA and BACTEC MGIT growth supplement (Becton Dickinson, Franklin Lakes, NJ, USA) following manufacturer’s instructions. BBL MGIT tubes were incubated for 42 days in a BACTEC MGIT 960 System. The remaining 5 ml were decontaminated in hexadecyl-pyridinium chloride 0.75% (w/v) for 12–18 h for solid culture. After a 5 min centrifugation step at 2,500 × *g*, pellets were cultured in Coletsos tubes (bioMérieux SAF-69280 Marcy l’Etoile France) at 37°C during 4 months.

Once the MGIT incubation period finished, DNA extraction was performed on culture from all positive tubes and some negative tubes. PCR was carried out subsequently to confirm that growth was due to *M. bovis* (21).

After the incubation period for solid culture was completed, colonies were visualized under a stereoscope and scraped for DNA extraction and *M. tuberculosis* complex PCR confirmation (21). All isolates were confirmed as *M. bovis* SB0339 by spoligotyping (22). A culture score was defined in order to categorize the infection level of each tissue. In this case, scores were as follows: 0: no growth, 1: less than 10 colonies, 2: between 10 and 50 colonies, and 3: over 50 colonies (20).

A tissue was considered positive for culture when it gave a positive result by solid culture, liquid culture, or both.

### Statistical Analysis

Reduction by vaccination was calculated by the formula (1 − VI/NVI) × 100 for each of the following parameters: solid culture score, number of positive tissues by culture, gross lesion score, and number of affected areas by gross pathology.

For the analysis, the number of tissues with positive cultures, with bTB compatible gross lesion and the solid culture and lesion scores, was calculated per area and animal. Differences in the degree of pathology and bacterial burden (lesion scores and culture scores) were compared using the Mann–Whitney *U*-test. Differences in the number of affected tissues by gross pathology and culture were assessed using Student’s paired two-sample *t*-test, whereas Fisher’s exact test was used for proportions of animals with lesions. Spearman’s correlation test was applied to assess the association between culture and gross pathology results. In all cases, significance of the differences among groups for all variables was accepted at *p* < 0.05.

## Results

### Clinical Signs

All animals included in the study went through the whole experiment, and no adverse reactions were reported after vaccination or challenge. No clinical signs of bTB such as wasting and coughing were observed in any of the animals after challenge. Infection of all challenged animals was confirmed by bacteriological and pathological analysis. The VNI group did not present gross lesions compatible with bTB or culture-positive tissues.

### Postmortem Analysis

Detailed pathology results from the infected groups of the study are shown in Table 1. The thoracic region and lung were the areas presenting a higher number of affected tissues and the NVI presented slightly higher scores as expected. Culture results from the infected groups of the study are shown in Table 2. In this case, again thorax and lung were the most affected areas, although the VI group only presented one animal with one positive tissue. The analysis has been focused separately on head, thorax, and lung as well as on the total where all areas of the animal have been considered. The analysis of number of tissues presenting pathology and culture positive results is shown in Table 3.

**Table 1:** Tissues with tuberculosis compatible lesions in the infected groups of the study.

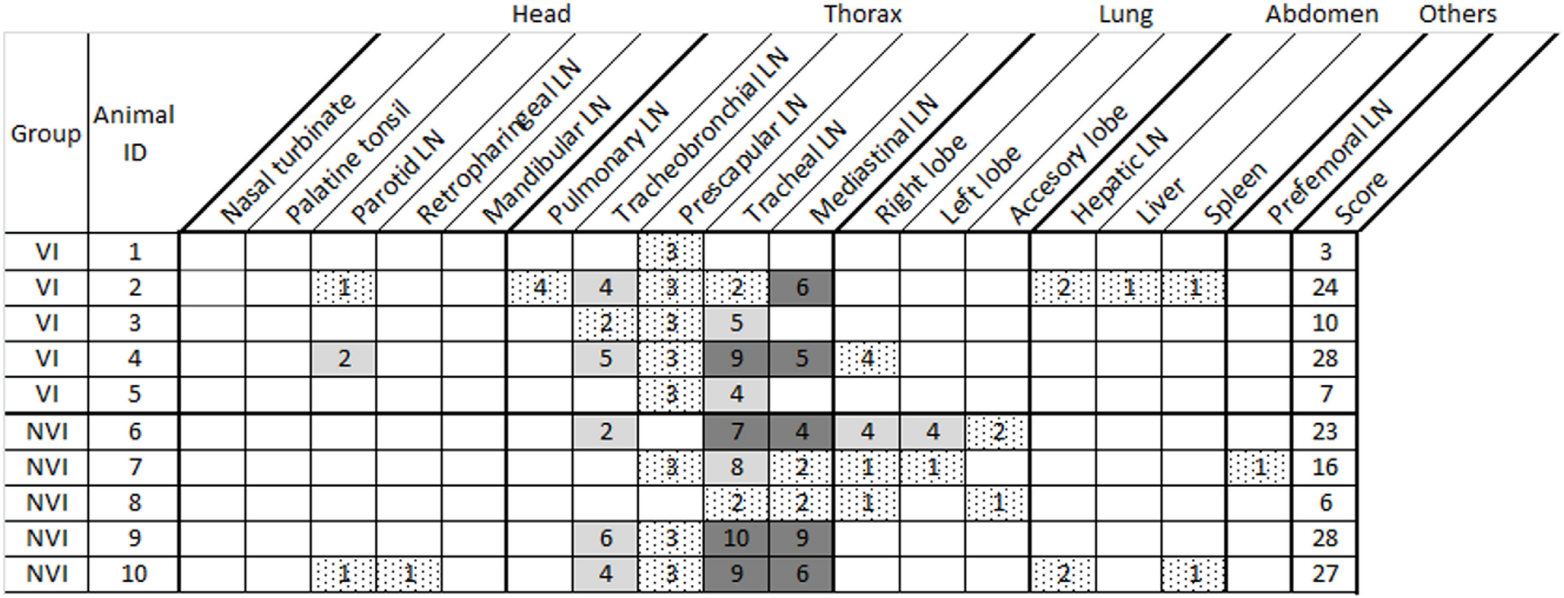

*VI, vaccinated infected; NVI, non-vaccinated infected; LN, lymph node*.

*One LN affected (right or left): dotted; two LNs affected (both right and left or two of three, cranial, caudal, or medial): light gray; three LNs affected (cranial, caudal, and medial): dark gray*.

*Lesion score is the sum of the scores of all tissues*.

**Table 2:** Tissues with positive culture results in the infected groups of the study.

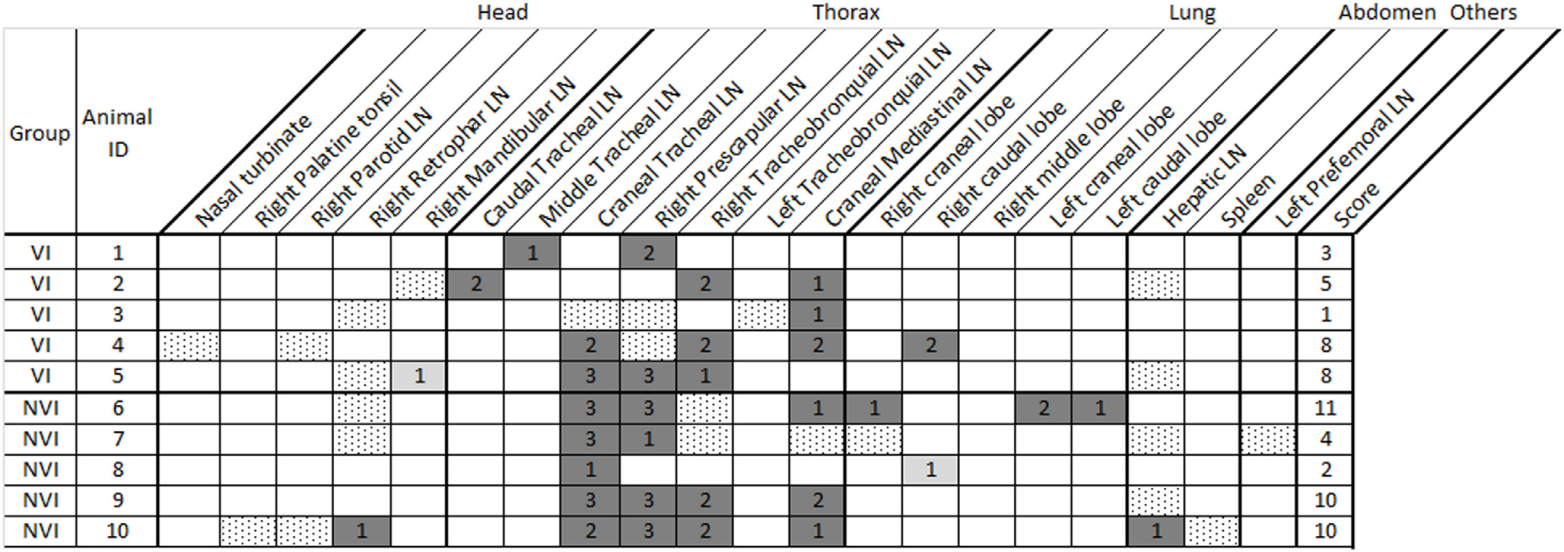

*VI, vaccinated infected; NVI, non-vaccinated infected; LN, lymph node*.

*MGIT culture positive: dotted; solid culture positive: light gray; both MGIT and solid culture positive: dark gray*.

*Culture score is the sum of the scores of all tissues*.

**Table 3:** Gross pathology and bacteriology analysis considering number of affected tissues per area.

	Pathology				Bacteriology			
	Mean (SEM)		*t-*Test		Mean (SEM)		*t*-Test	
		
	NVI	VI	*p*	% R	NVI	VI	*p*	% R
Head	0.4 (0.40)	0.6 (0.4)	0.733	0.00	1.0 (0.55)	1.4 (0.4)	0.572	0.00
Thorax	6.4 (1.43)	5.4 (1.50)	0.643	15.62	3.4 (0.60)	3.4 (0.40)	1.000	0.00
Lung	1.8 (0.92)	0.2 (0.20)	0.126	89.00	1.0 (0.55)	0.2 (0.20)	0.207	80.00
Total	9.2 (1.74)	6.8 (2.35)	0.436	26.10	6.6 (1.40)	5.4 (0.93)	0.495	18.18

*NVI, non-vaccinated infected; VI, vaccinated infected; R, reduction due to vaccination*.

In the thoracic region, the NVI group presented a mean of 6.4 LNs with gross lesions with a minimum of two and a maximum of 10 affected LNs in each animal compared to the VI group with an average of 5.4 LNs affected with a minimum of two and a maximum of nine affected LNs. Significant differences among groups were not observed (*p* = 0.643). However, the reduction due to vaccination was of 15.62%.

Of the six lung lobes evaluated, the NVI group presented an average of 1.8 lobes with gross lesions with a minimum of zero and a maximum of five affected lobes per animal, much higher than the mean of 0.2 affected lobes found in the VI group, which had a minimum of zero and a maximum of one affected lobes. Although significant differences were not observed between the number of affected lobes, the tendency should be considered (*t*-test, *p* = 0.126). This represents an 89% reduction due to vaccination. Only one animal (1/5, 20%) from the VI group presented lesions in the lung compared to three animals (3/5, 60%) from the NVI group (Fisher’s test; *p* = 0.189).

The number of affected tissues was always lower in the VI group than in the NVI groups except for the area that compromised the head. In this case, two animals presented gross lesions, one in one tissue and another in two in the VI group, whereas the NVI group presented one animal with gross lesions in two tissues. Tissue culture positivity was also slightly higher in the head in the VI group where an average of 1.4 ± 0.4 tissues with detectable bacteria was observed compared to the mean of 1 ± 0.55 tissues in the NVI group. In the VI group, four animals presented positive tissue culture (4/5, 80%) compared to three in the NVI group (3/5, 60%).

**Figure 1:**
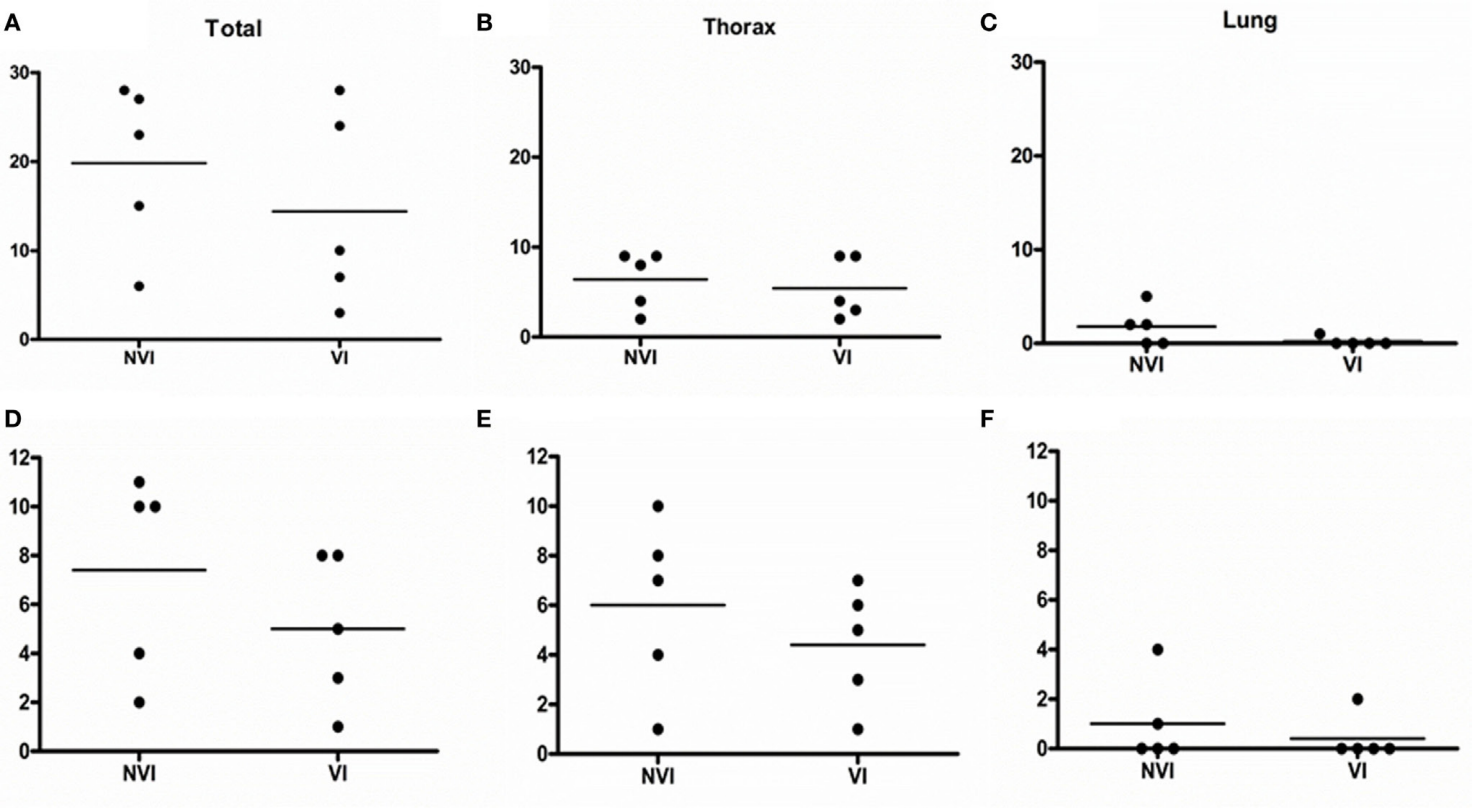
Bovine tuberculosis lesion and culture scores for total **(A,D)**, thorax **(B,E)**, and lung **(C,F)**. The solid lines present median values.

Pathology and culture scores were always lower in the VI group than in the NVI group (Table 4 and Figure 1) except for the head where lesion scores were slightly higher in the VI group (0.6 ± 0.4 vs. 0.4 ± 0.4). In any case, significant differences were not observed among analyzed areas. However, reductions in lung of 71.42 and 60% in lesion and culture scores, respectively, and of 23.75 and 26.66% in the vaccinated group for the same parameters in thorax should be noted.

**Table 4:** Gross pathology and bacteriology analysis considering lesion and culture score per area.

	Pathology				Bacteriology			
	Mean (SEM)		*U-*test		Mean (SEM)		*U*-test	
	
	NVI	VI	*p*	% R	NVI	VI	*P*	% R
Head	0.4 (0.40)	0.6 (0.40)	0.606	0.00	0.2 (0.20)	0.2 (0.20)	1.000	0.00
Thorax	16.0 (4.13)	12.2 (3.60)	0.401	23.75	6.0 (1.58)	4.4 (1.07)	0.344	26.66
Lung	2.8 (1.85)	0.8 (0.80)	0.288	71.42	1.0 (0.77)	0.4 (0.40)	0.521	60.00
Total	20.0 (4.10)	14.4 (4.90)	0.530	28.00	7.4 (1.83)	5.0 (1.37)	0.248	32.43

*NVI, non-vaccinated infected; VI, vaccinated infected; R, reduction due to vaccination*.

Correlation between both diagnostic methods considering number of affected tissues was best fit in lung (*r* = 0.988, *p* < 0.0001), followed by head (*r* = 0.746, *p* = 0.013), total (*r* = 0.667, *p* = 0.035), and thorax (*r* = 0.655, *p* = 0.04).

## Discussion

Although the efficacy of PTB vaccination has been repeatedly demonstrated (7, 11, 18, 23), its use has been restricted due to the cross-reactivity with current bTB diagnostic methods. Interference issues have probably led to the lack of knowledge on the effect of PTB vaccination in the development of bTB infection. This is the first study examining this topic in bovines under experimental conditions.

In this particular experimental setting, all animals became infected regardless of their vaccination status showing positive culture results and macroscopic lesions compatible with bTB. In both groups, thorax and lung were the most affected areas. The majority of tissue sites affected by infection in terms of lesion development or bacterial colonization, as well as the highest lesion and culture scores appeared in these two areas. However, PTB vaccination seemed to induce a moderate protective response against *M. bovis* challenge, which led to a reduction of the pathological and bacteriological results in both areas of the VI group. The effect of vaccination was most evident in the lung as seen by the fact that only one animal (1/5, 20%) of the VI group presented lesions and detectable bacteria in this area compared to the NVI group where three animals were affected (3/5, 60%). Same tendency was observed in the remaining studied areas except for the head, where the protective effect was not evident. Although differences among groups were not statistically significant and only tendencies have been observed, the degree of protection observed can be considered biologically relevant. To reinforce the appealing results obtained from this first trial, it would be necessary to carry out further studies with a larger sample size.

These results suggest that a certain degree of heterologous protection against *M. bovis* infection takes place after PTB vaccination, and although the protection conferred is probably not enough to impede the establishment of the disease or prevent horizontal transmission within a herd, it may contain the infection to some extent. Results from this experiment indicate that this containment would clearly benefit the lung (60–89% reduction in lesions and bacterial burden) since reduction due to vaccination in the thoracic region was less notorious and absent in the head area. This may be important considering that the main excretion route of *M. bovis* is through the respiratory system, and therefore, the reduction of the bacterial load in the lung may lead to a reduction of this figure in the environment.

These findings partially agree with the results obtained by Pérez de Val et al. (14) in goats after PTB vaccination and subsequent challenge with *M. bovis* where, lesions in vaccinated goats appeared only in the lung and corresponding LNs whereas non-vaccinated animals showed an increased dissemination frequency of the lesions. In that case (14), goats were challenged through the endobronchial route and, therefore, infection progressed mainly affecting the lower respiratory tract. In another study in goats in which the transthoracic route was used, lesions were mainly located in lung and mediastinic and mesenteric LNs (24). In our study, thorax was the primary focus and extrathoracic and extrapulmonary dissemination of bacteria to the upper respiratory tract or head area (retropharyngeal, mandibular, parotid LNs, and nasal turbinate) occurred in four animals of the VI group and three animals of the NVI group. This could be due to a pulmonary dissemination to the head by mycobacterial shedding in the tracheobronchial secretions and subsequent ingestion as hypothesized in previous reports (25). Bacteria and gross lesions were detected in spleen of one animal of the NVI group, whereas only gross lesions were observed in spleen and liver of one animal of the VI group indicating that systemic circulation of mycobacteria had occurred.

Added to the route, the challenging dose can be crucial for the pathological outcome of the infection. In previous studies in goats (14, 24, 25), animals were challenged with lower doses of *Mycobacterium caprae* (10^2^–1.5 × 10^3^ CFUs), but as stated before in those studies, the inoculum was directly deposited into the lung. The selected dose (10^6^ CFU) and infection route (endotracheal) may be responsible for the wider spreading of the lesions in our study. This high dose was applied to guarantee infection for vaccine evaluation. In any case, considering the fact that in experimental conditions the bacterial load administered for challenge is most probably many logs higher than the amount of *M. bovis* that an animal will be in contact with in field conditions it could be expected that higher protection levels would be observed in these cases.

Positive correlations were found between pathological and bacteriological techniques as expected. These were best fit in the lung, area that poses the most noticeable partial protective effect by the vaccine.

The results presented here suggest that vaccination against PTB modifies the course of experimental bTB infection by decreasing the severity of the lesions and the bacterial burden. Although our results are not conclusive, they support the view that mycobacterial vaccines could potentially be useful tools for disease control in specific settings where vaccination does not interfere with eradication programs.

## Ethics Statement

With the purpose of obtaining data for this trial, calves were submitted to clinical practices standardized and regulated by the European, Spanish and Regional Law and Ethics Committee. The experimental design underwent ethical review and approval by NEIKER’s Animal Care and Use Committee and by the Agriculture Department (PARAPATO-1264-BFA).

## Author Contributions

RJ, JG, and IS conceived the study. MS, EM, MG, IS, and NE carried out the laboratory work. RJ, NE, and MS compiled and analyzed the data. MS, NE, and IS collated the results. MS, NE, IS, and JG drafted the preliminary manuscript. All authors participated in the review and the editing of the final draft and also read and approved its final version.

## Conflict of Interest Statement

The authors declare that the research was conducted in the absence of any commercial or financial relationships that could be construed as a potential conflict of interest.
